# Reach Space Analysis of Baseline Differential Extrinsic Plasticity Control

**DOI:** 10.3389/fnbot.2022.848084

**Published:** 2022-06-01

**Authors:** Simon Birrell, Arsen Abdulali, Fumiya Iida

**Affiliations:** Bio-Inspired Robotics Laboratory, Department of Engineering, University of Cambridge, Cambridge, United Kingdom

**Keywords:** Differential Extrinsic Plasticity, self-organization, robotic control, play, intrinsic motivation, neuroplasticity, reinforcement learning, complexity

## Abstract

The neuroplasticity rule Differential Extrinsic Plasticity (DEP) has been studied in the context of goal-free simulated agents, producing realistic-looking, environmentally-aware behaviors, but no successful control mechanism has yet been implemented for intentional behavior. The goal of this paper is to determine if “short-circuited DEP,” a simpler, open-loop variant can generate desired trajectories in a robot arm. DEP dynamics, both transient and limit cycles are poorly understood. Experiments were performed to elucidate these dynamics and test the ability of a robot to leverage these dynamics for target reaching and circular motions.

## 1. Introduction

Robot control is still very much a work in progress. While much has been learned of how humans and animals control their bodies (Winter, 2009), either outright or after a learning process, we still do not know enough to be able to design a robot that even approaches human dexterity. Classical control theory and more recently Reinforcement Learning (RL) have been extensively studied but are still subject to lack of robustness, the curse of dimensionality and unreasonably high learning times (Sutton and Barto, 2018).

One issue with these frameworks is the assumption that the brain directly controls the output of each available degree of freedom; typically a learning agent will adjust its body's motor torques at each time step to produce a desired result in a rigid body system within a given environment (see for example OpenAI Gym; Brockman et al., 2016). This is clearly not how biology tackles the problem. In a human, descending signals from the cortex pass through and are modified by interneurons with their own neuroplasticity mechanisms, which activate bundles of muscle fibers and drive an underactuated soft body with extremely complex dynamics (Pierrot-Deseilligny and Burke, 2005; Winter, 2009). On the face of it, we have no hope. If we can't reliably control a mathematically much simpler rigid robot, how could we possibly control an agent with a similar complexity to a human body?

On the other hand, could the complexity of the human body actually be a help and not a hindrance to the perception/control problem? The bio-inspired research agenda known as Embodied Intelligence suggests so (Pfeifer and Bongard, 2006; Cangelosi et al., 2015) and has spawned many different initiatives in this area. One approach is morphological computation, which investigates the ways that parts of the information processing burden can be offloaded to the body itself (Hauser et al., 2012; Müller and Hoffmann, 2017), through sensor morphology (as in the case of flies' eyes) (Iida and Nurzaman, 2016) or through the simplification of control (Eder et al., 2018). Physical reservoir computing uses the complexity of the body for general purpose computation (Nakajima, 2020). The richness of behavior of the peripheral nervous system, providing fast-acting reflexes and hierarchical and coordinated control (Côté et al., 2018) has been less applied to robotic research, which almost exclusively models the control problem as the agent's brain directly driving motor torques. Finally, investigations into different neuroplasticity schemes show that a surprising variety of complex, environmentally-aware behaviors can be spontaneously generated from simple, biologically plausible neuroplasticity rules within sensorimotor loops (Zappacosta et al., 2018).

These latter neuroplasticity-generated spontaneous behaviors, detailed in the book “The Playful Machine” (Der and Martius, 2012) and in related papers (Der and Martius, 2015, 2017), can drive simulated agents to explore and react to their environments in a manner that is highly suggestive of natural behaviors without building in any goals or higher-level planning of any sort. The most recent iteration of this research uses a particular neuroplasticity scheme called Differential Extrinsic Plasticity (DEP) (Der and Martius, 2015; Pinneri and Martius, 2018) to generate intriguing behaviors that are tightly coupled with the environment: a four-legged creature will appear to search for and find ways to climb over a fence; a humanoid will eventually clamber out of a hole it is trapped in. From our external observer perspective, these embodied behaviors appear to be goal-driven, but yet they are not. DEP has emerged as an interesting and promising candidate plasticity rule, but to date no practical applications for it have yet been found. It is an autonomous goal-free controller rather than a useful control method or component in a larger system.

A complicating factor in the practical usage of DEP is the current lack of an analytical solution, despite the research time invested. In all likelihood, even simple DEP systems are too complex to be fully described analytically and so research has tended to be empirical, treating DEP as a pre-existing natural phenomenon. This is not an insurmountable issue; it places DEP within the context of related research into algorithmic information and complexity theory, both areas cited in theories of the development of the human brain (Hiesinger, 2021). The behavior of DEP may not be solvable analytically even if it is deterministic. It may be undecidable: the only way to determine the output being to run it in simulation.

Given this, how can we study DEP and map out its potential? First, we must simplify: by temporarily removing environmental feedback we can map out baseline behaviors for DEP, following the methods employed in Pinneri and Martius (2018). Second, we must test the control-ability and limits of what DEP can do: to what extent can higher order systems “request” particular behaviors, and how much coverage will these behaviors provide in the context of a given task?

This paper takes the first steps in this direction. By employing “short-circuit DEP” (see below) and with a simple test case, where the output of short-circuit DEP drives a simulated 2 degree of freedom (DOF) robot arm, we show that DEP can be made to accomplish specific goals and that these goals cover a useful region of task space.

## 2. Materials and Methods

### 2.1. How “Classical” Differential Extrinsic Plasticity Works

**Figure 1:**
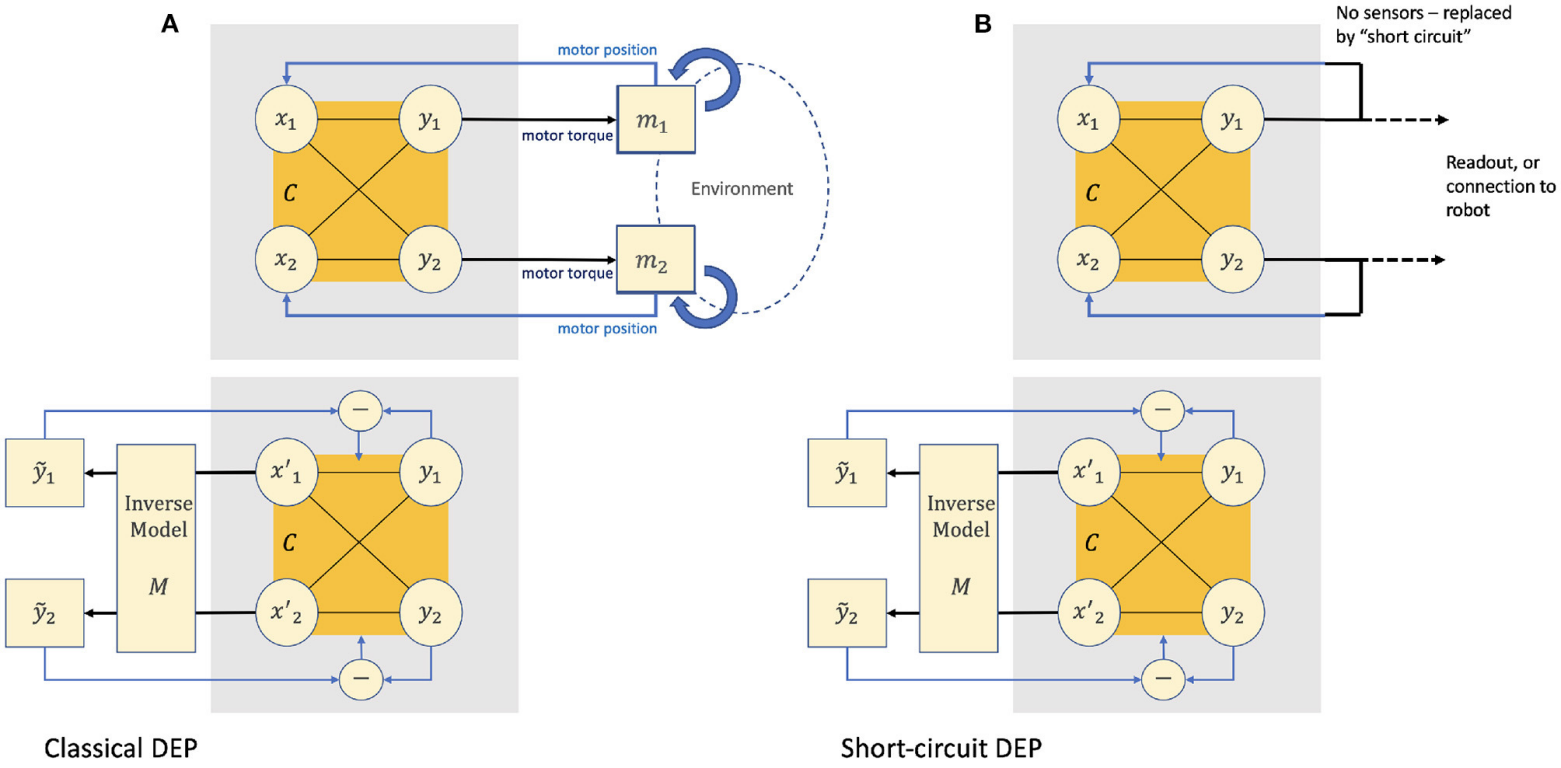
In the “classical” version of Differential Extrinsic Plasticity (DEP) comprises two overlapping dynamical systems. [**(A)**, top] The input layer *x* of a feed-forward neural network *C* is driven by the motor positions. The output layer *y* drives the motor torques. These motors operate on the agents body in a given environment (potentially with collisions) and the resulting motor positions *x*^*t*+1^ will be fed back to the input to start the cycle again. [**(A)**, bottom] The positional information returned as *x*^*t*+1^ is also fed to an inverse model that infers the rate of change of the motor torques $\tilde{ẏ}$ that would have generated them in the absence of environmental feedback. The difference between $\tilde{ẏ}$ and the actual rate of change ẏ captures the environmental effects of the agent's actions. This difference is then used to modify the weights of C. In the “short-circuited version” of DEP [**(B)**, top and bottom] there are no motors or sensors; the output *y* of the network is fed directly back to the input *x*.

DEP describes a way of wiring motors and related sensors together with a neuroplasticity rule, such that a DEP-enabled agent produces a large set of “natural looking” behaviors that respond to interactions with the environment. Summarizing (Der and Martius, 2015; Pinneri and Martius, 2018), this section describes the equations that define the thermoplasticity rule for the “classic” version of DEP (see Figure 1A).

For a two-layer artificial neural network with input layer *x*_*i*_, output layer *y*_*i*_, weights *C*_*ij*_, biases *h*_*i*_, and a *tanh* activation function, the output activation is given by:


(1) $$\begin{array}{l} y_{i}=tanh\left(\overset{n}{\underset{j=1}{\sum }}C_{ij}x_{j}+h_{i}\right) \end{array}$$

A simple feedback controller for an agent with rotary motors may then be constructed where *y*_*i*_ drives motor torques and *x*_*i*_ is driven by the resulting motor positions (see Figure 1A for a 2 degree of freedom example). In itself, this is not a very interesting controller, although given that the motor positions are ultimately determined not only by the applied torques but also by the body in which they're embedded and its interaction with the environment, nor is it trivial. This neural controller, the body and the environment together form a single dynamical system.

The behavior of this dynamical system can be overlaid by a second dynamical system driven by neural plasticity, that is, the evolution over time of the controller's weights. Many plasticity schemes have been studied (see Table 1). Hebbian learning modifies the weights based on the product of pre and post-synaptic activations^1^. Differential Hebbian Learning is similar (Zappacosta et al., 2018), but uses the product of the rates of change of the two activations.

**Table 1:** Different plasticity schemes.

**Plasticity scheme**	**Update rule**
Hebbian learning	τĊ_*ij*_ = *x*_*i*_*y*_*j*_
Differential Hebbian learning	τĊ_*ij*_ = ẋ_*i*_ẏ_*j*_
Differential extrinsic plasticity	τĊ_*ij*_ = ẋ_*i*_(ẏ_*j*_+δẏ_*j*_)−*C*_*ij*_

Differential Extrinsic Plasticity extends Differential Hebbian learning by introducing an inverse model *F* that maps the rate of change of received sensor values ẋ^*t*+1^ back to the inferred rate of change of motor torques ${\tilde{ẏ}}^{t}$ that caused them:


(2) $$\begin{array}{l} {\tilde{ẏ}}^{t}=F\left({ẋ}^{t+1}\right) \end{array}$$

In most DEP implementations the inverse model *F* is implemented as a simple matrix *M* such that


(3) $$\begin{array}{l} {\tilde{ẏ}}^{t}=M{ẋ}^{t+1} \end{array}$$

and, as in this paper, it is often assumed to be the identity matrix. *F* isn't required to be strictly accurate to reproduce DEP's behavior (Der and Martius, 2015).

The revised update rule uses $\tilde{ẏ}$ in place of ẏ and adds a damping term. Dropping the time superscript *t*:


(4) $$\begin{array}{l} \tau {Ċ}_{ij}={ẋ}_{i}{\tilde{ẏ}}_{j}-C_{ij} \end{array}$$

One way to think about $\tilde{ẏ}$ is as the sum of the real historical value for ẏ at *t* plus an error term δẏ with respect to the model *F*.


(5) $$\begin{array}{l} \tilde{ẏ}=ẏ+\delta ẏ \end{array}$$

This substitution is shown in the final row of Table 1. Comparing it to the scheme for Differential Hebbian Learning shows how this “unexpected” environmental feedback is incorporated into the weight updates.

The weight matrix *C* is normalized to Ĉ at each time step with a factor κ and a parameter ρ that prevents a division by zero.


(6) $$\begin{array}{l} Ĉ\leftarrow \kappa C/\left(\|C\|+\rho \right) \end{array}$$

Finally, the activation rule is modified from Equation (1) to use the normalized Ĉ rather than *C*:


(7) $$\begin{array}{l} y_{i}=tanh\left(\overset{n}{\underset{j=1}{\sum }}{Ĉ}_{ij}x_{j}+h_{i}\right) \end{array}$$

The combination of these two overlaid dynamical systems produces an agent that cycles through a series of complex behaviors that are responsive to environmental feedback.

### 2.2. How “Short-Circuit” DEP Works

A simplified version of DEP was used in Pinneri and Martius (2018) for an empirical analysis of its behaviors. In this configuration there are no motors or sensors; the system output *y* is connected directly back to the system input *x* (Figure 1B). As ${\tilde{ẏ}}^{t}=M{ẋ}^{t+1}$ and ẋ^*t*+1^ = ẏ^*t*^ and *M* is the identity matrix the update rule simplifies to


(8) $$\begin{array}{l} \tau {Ċ}_{ij}={ẋ}_{i}{ẏ}_{j}-C_{ij} \end{array}$$

This is effectively Differential Hebbian Learning with damping and normalization.

**Figure 2:**
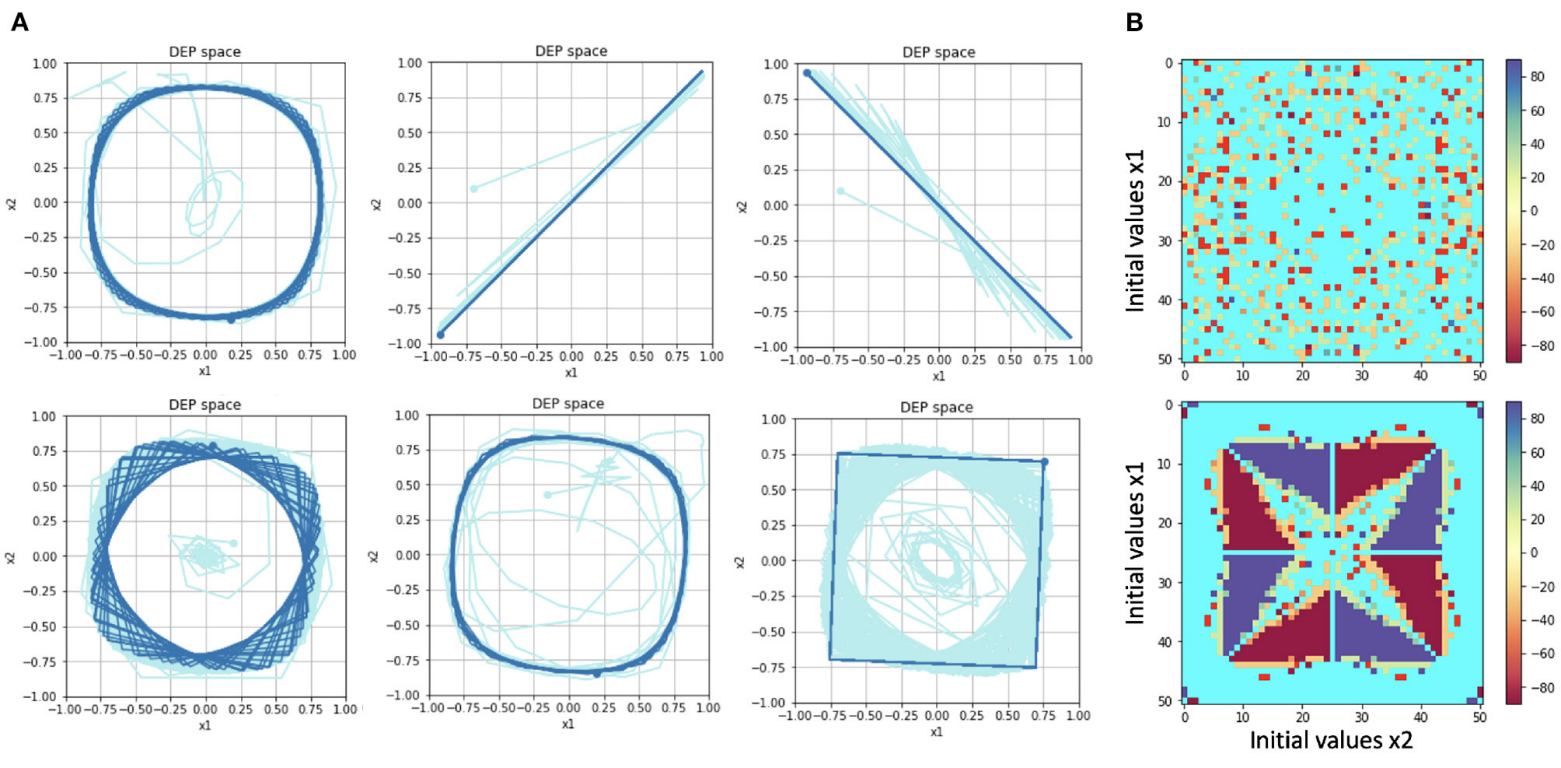
**(A)** Examples of limit cycles reached by “Short-circuit” DEP. The phase diagrams show the trajectory of the system along the dimensions *x*_1_ and *x*_2_. The second and third trajectories oscillate between two endpoints. The other trajectories are all rotational. **(B)** Maps of the attractors reached based on initial values of *x*_1_, *x*_2_. The color bar refers to the rotational angle of the attractor, in the case of rotational attractors. Cyan refers to the non-rotational attractors shown in the second and third examples in **(A)**. The resulting map is shown in the top row. The map in the lower row reproduces the one shown in Pinneri and Martius (2018), but to generate it requires slightly altering the DEP algorithm (see text). Our version lacks their basins of attraction.

By eliminating the environment, the behaviors generated can be simplified to a set of predictable limit cycles, examples of which are shown in Figure 2A. The limit cycles reached depend on the initial conditions of the system, in particular the initial values for $x_{1},x_{2},y_{1},y_{2},\dot{x_{1}},\dot{x_{2}},\dot{y_{1}},\dot{y_{2}}$, and *C*. In Pinneri and Martius (2018), all initial values were held constant except for *x*_1_, *x*_2_.

Following that paper, a map of the attractors reached based on differing initial conditions for *x*_1_, *x*_2_ is shown in Figure 2B. For each fixed point, the final 2x2 Ĉ matrix, is considered to be a rotational matrix and the corresponding angle is assigned a color. There are two cases of non-rotational matrices: a zero matrix (which is assigned bright red) and period-2 oscillations, such as the second and third examples in Figure 2A, which are assigned cyan. The resulting map is shown in Figure 2B (top).

It should be noted that (Pinneri and Martius, 2018) obtained a different pattern, as that paper used code that inadvertently reset the *C* matrix to zero at *t* = 2, generating different dynamics (private communication). Their results were reproduced (with the necessary code modification) in Figure 2B (bottom). For our experiments we followed the strict interpretation of the DEP equations. The attractors identified are the same, but the attractor map with respect to initial conditions is different; the “basins of attraction” cited in that paper being absent. In our opinion, these basins are an artifact of the previous code base and not intrinsic to DEP as such.

In the present paper's experiments, as well as *x*_1_, *x*_2_, the initial value *C*_0_ of the matrix *C* is also varied. It was discovered that choosing different values for *C*_0_ elicits different trajectories and ultimate limit cycles for each combination of the initial values *x*_1_, *x*_2_. One way of looking at this is to say that different *C*_0_ can select different behaviors for a given initial *x*_1_, *x*_2_.

### 2.3. The Experimental Setup

**Figure 3:**
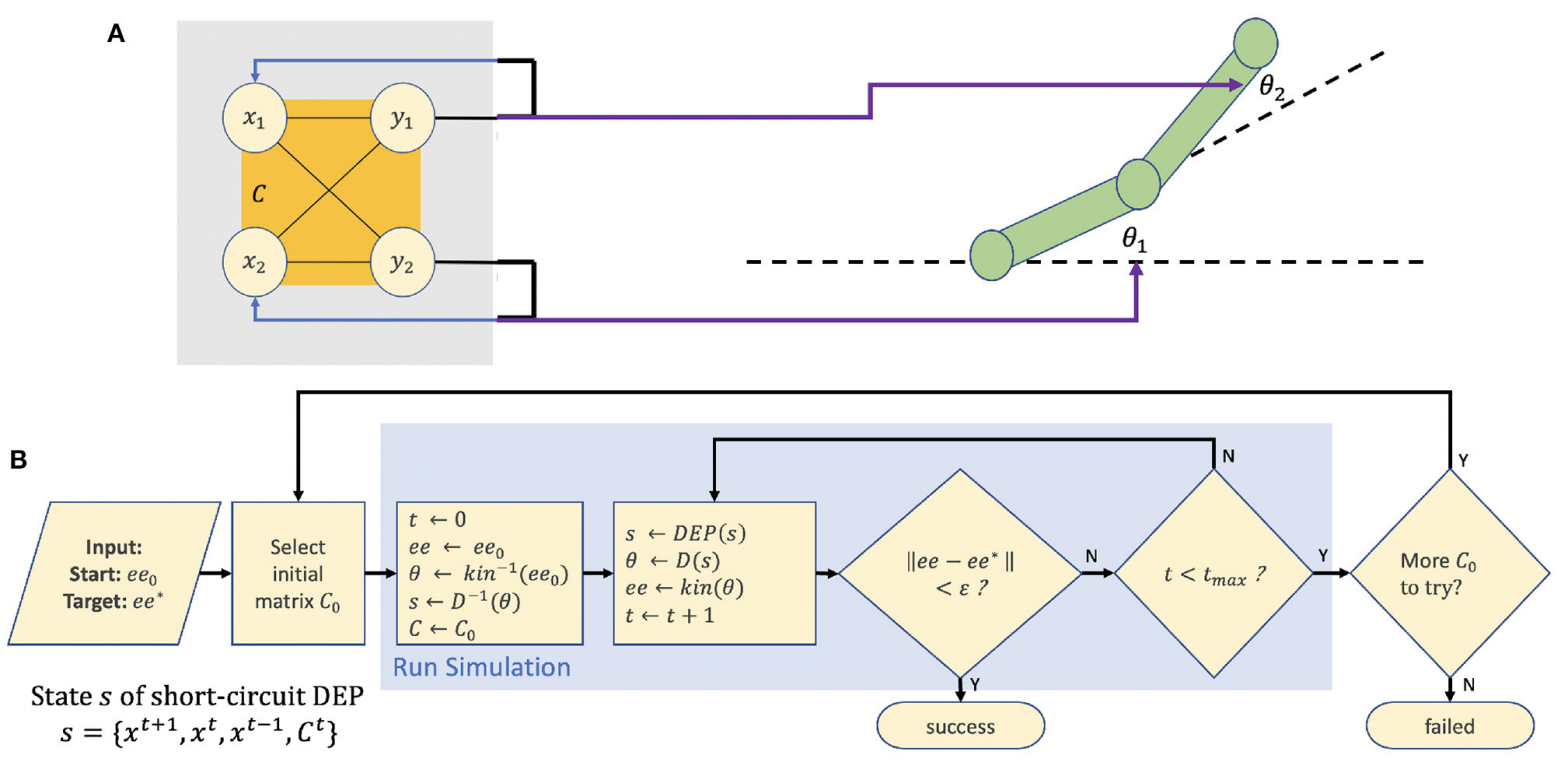
**(A)** The experimental setup. A “short-circuit” DEP controller drives a 2DOF robotic arm in an open loop fashion. **(B)** A flowchart of the search algorithm for obtaining a value for *C*_0_ that reaches a desired target ee^*^ from starting position *ee*_0_.

In the two experiments described, the “short circuit” DEP system is used to drive a simple 2 degree of freedom robotic arm (see Figure 3A).

The state *s* of the short-circuit DEP system can be fully captured as


(9) $$\begin{array}{l} s=\left\{x^{t+1},x^{t},x^{t-1},C^{t}\right\} \end{array}$$

so that at each timestep *s*^*t*+1^ ← *DEP*(*s*^*t*^).

We can then use a robot arm with segment lengths *l*_1_, *l*_2_, here 0.5 m, to “read out” the state *s* of DEP. The joint angles θ, comprising θ_1_, θ_2_, are driven by a “driver” function *D* that is specific to a given task type, such that


(10) $$\begin{array}{l} \theta =D\left(s\right) \end{array}$$

Note that this is an open-loop controller. None of the reported benefits of environmentally-aware “Classic” DEP are used here, in line with the goal of learning to control a very simple DEP system. The position of the robot's end effector can be considered as a simple transformation or readout of DEP's internal state *s*.

Two types of task are considered. In the first, the goal is for the robot arm's end effector that starts at position *ee*_0_ to **reach** an arbitrary target position *ee*^⋆^. For this type of task, function *D*(*s*) = *D*_*reach*_(*s*) is simply


(11) $$\begin{array}{l} \theta =D_{reach}\left(\left\{x^{t+1},x^{t},x^{t-1},C^{t}\right\}\right)\\ \;=\pi x^{t+1}\\ \;=\pi y \end{array}$$

In other words, the output of *y* of short-circuit DEP directly drives the motor angles θ.

**Figure 4:**
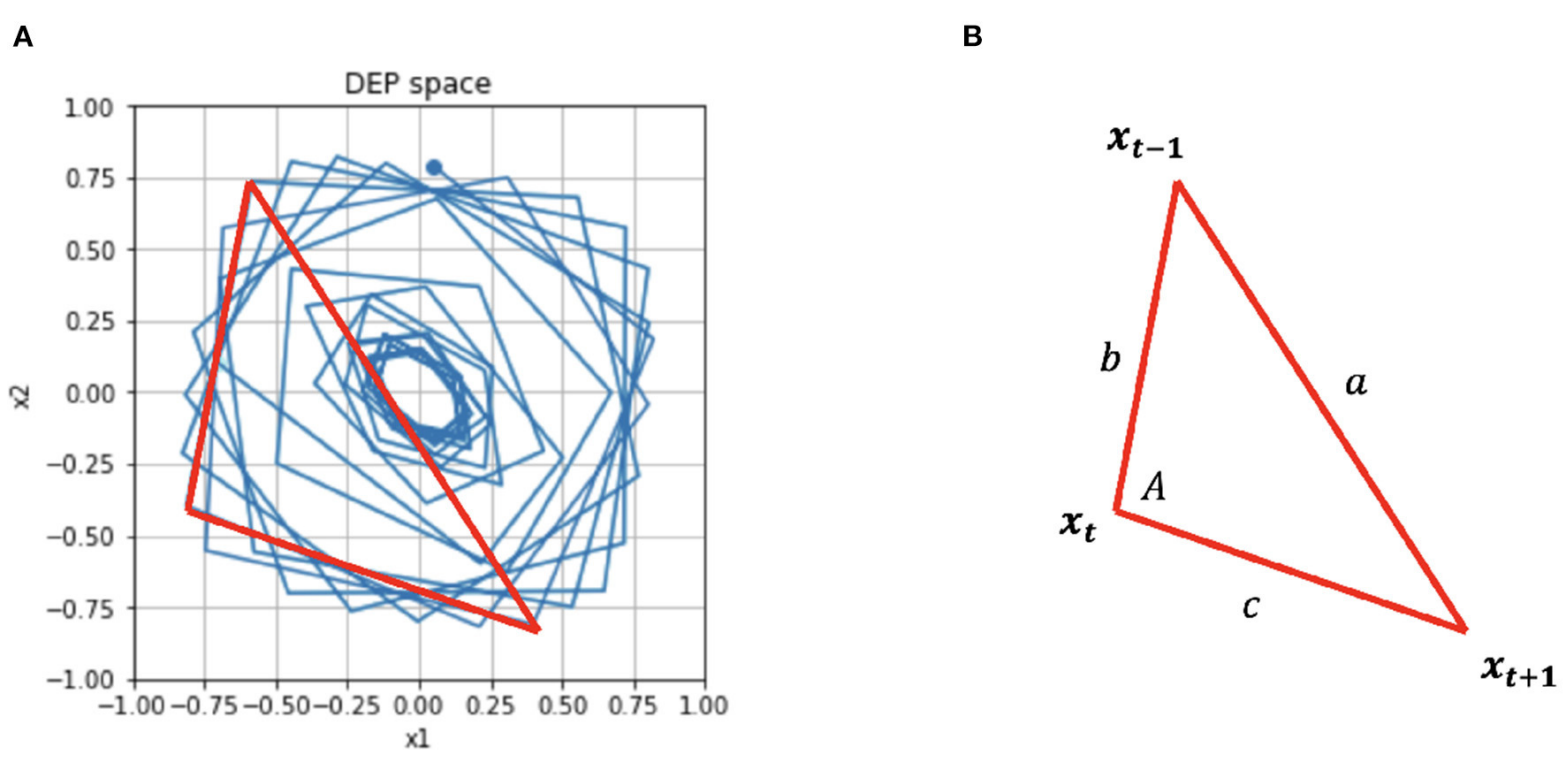
**(A)** For the circular controller, θ_2_ is derived from the angle between subsequent vertices of *x*. **(B)** This angle A is calculated by simple geometry.

In the second type of task, the goal is for the end effector to trace a **circular** trajectory of arbitrary radius *r*. Here, *D*(*s*) = *D*_*circle*_(*s*) and we leverage the angle *A* between the vectors *x*^*t*+1^−*x*^*t*^ and *x*^*t*^ − *x*^*t*−1^. See Figure 4 for the simple geometry that defines *a, b, c*. Then, the two joint angles θ_1_, θ_2_ can be defined in the new driver function:


(12) $$\begin{array}{l} \begin{array}{c} \theta =D_{circular}\left(\left\{x^{t+1},x^{t},x^{t-1},C^{t}\right\}\right)\\ \theta_{i}=\{\begin{array}{cc} \omega t & \text{if }i=1\\ cos^{-1}\left(\frac{b^{2}+c^{2}-a^{2}}{2bc}\right) & \text{if }i=2 \end{array} \end{array} \end{array}$$

At a fixed point of *C*, Ĉ^*t*+1^ → *C*^*t*^ and if |*x*| << 1,


(13) $$\begin{array}{l} x^{t+1}=tanh\left(Ĉx^{t}\right),\\ \;x^{t+1}\approx Ĉx^{t} \end{array}$$

Under these conditions, *x* is rotating around the origin in DEP space and the angle between every other point is approximately constant. As this angle drives θ_2_ then θ_2_ will also be a constant. $\dot{\theta_{1}}$ is a constant, so the robot will describe a circle.

### 2.4. The Search Algorithm for *C*_0_

Given an input of an initial end effector position *ee*_0_ and target position or trajectory *ee*^⋆^, our goal is to obtain an initial matrix *C*_0_ that will drive the system to reach *ee*^⋆^. *C*_0_ is obtained by a search algorithm, detailed in Figure 3B.

The 2 × 2 matrix *C*_0_ has four parameters that here each vary between −1 and +1. The algorithm linearly divides the range of each parameter into eight values, giving 8 × 8 × 8 × 8 = 4,096 possible values for *C*_0_. A simple grid search is performed, with each value being trialed in a rollout of 20,000 time steps.

In the case of the reaching task, at each time step of the rollout, if the distance between *ee*_*t*_ and *ee*^⋆^ is within a given tolerance ϵ, then success is declared. 10 random starting positions *ee*_0_ and 10 random targets *ee*^⋆^ were combined to give 100 trials, each of which is an execution of the algorithm in Figure 3B.

In the case of the circular task, success is declared after a full rotation of the end effector, where the mean squared radius error with respect to *r* is less than ϵ. Five random starting positions *ee*_0_ and five random radii *r*^⋆^ were combined to give 25 trials, each of which is an execution of the algorithm in Figure 3B.

The experiments were implemented in Python on Jupyter notebooks. The full source code may be downloaded from GitHub^2^, inspected and run.

## 3. Results

The trajectories in DEP space produced in the experiments generally consisted of a transient phase where the system “wanders” in *x*_1_, *x*_2_ followed by a limit cycle phase. The Reaching task leveraged both transient and limit cycle phase, while the Circular task leveraged the limit cycles.

### 3.1. The Reaching Task

One hundred trials of the Reaching task were performed. In every case, the system reported success: it found a path to all end effector targets from all end effector starting positions. The tolerance ϵ| had a value of 0.01 m.

**Figure 5:**
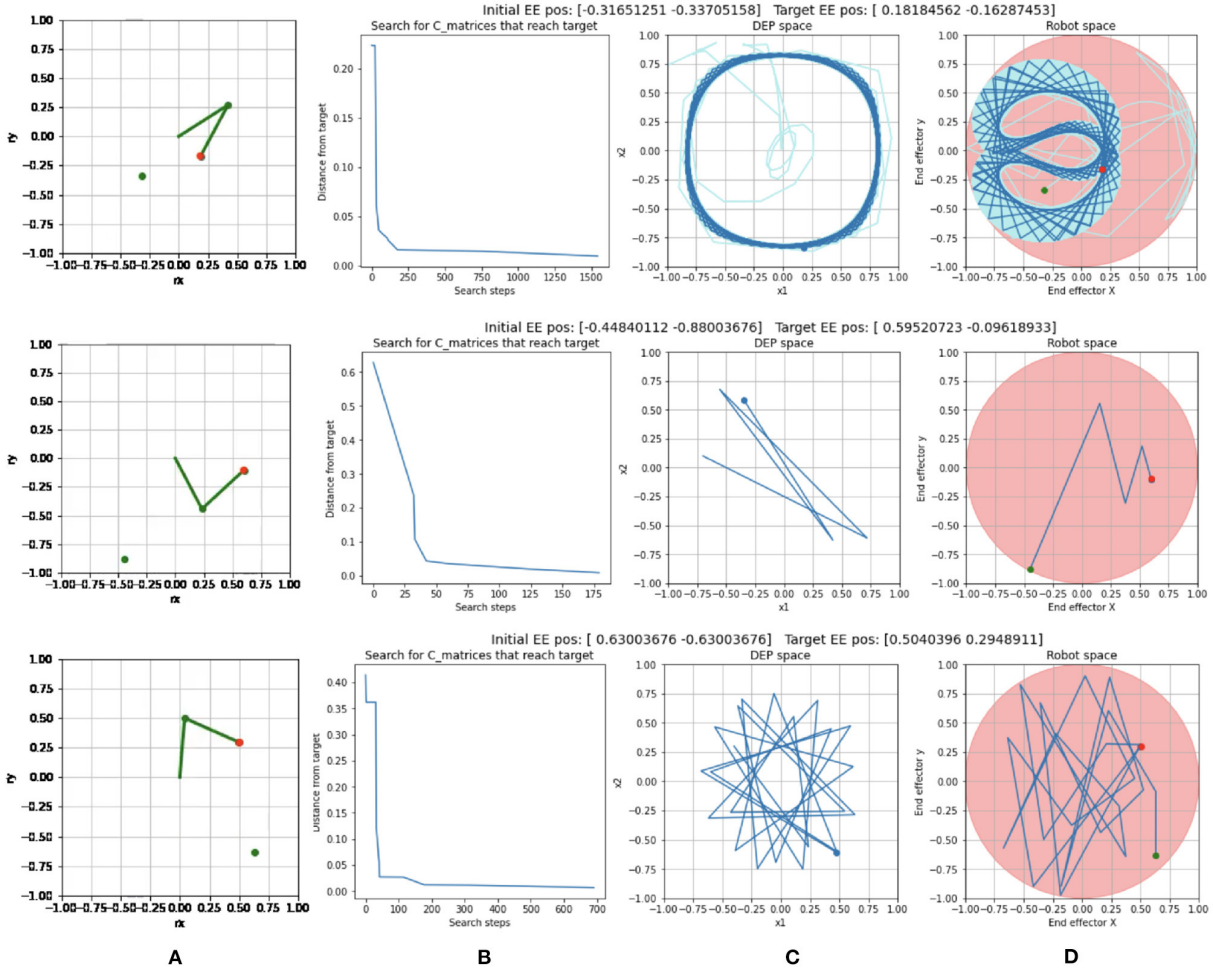
Examples of Reaching trials with: **(A)** different end effector start positions (green dot) and target positions (red dot). **(B)** The progress of the search for a solution C matrix. **(C)** The solution trajectory in DEP space for the successful trial. **(D)** The solution trajectory in robot space for the successful trial. The most recent positions are shown in dark blue while the early part of the trajectory is in light blue.

Trajectory examples are show in Figure 5. In the example in the top row, the search algorithm tested 1,541 *C*_0_ matrices (Figure 5B) before finding a value that caused the end effector (Figure 5A) to reach the desired target. The solution itself in DEP space (Figure 5C) and robot space (Figure 5D) show that the system had entered a rotational limit cycle before reaching the target.

A second example, in the second row of Figure 5 shows a contrasting example where a solution was found after testing only 179 search steps. In the solution, the system was still in a transient phase when it hit the target, at only six time steps into the rollout.

### 3.2. The Circular Trajectory Task

Twenty-five trials of the Circular task were performed. In every case, the system reported success: it managed to describe a circular trajectory of at least one rotation where the mean squared radius error with respect to the desired radius was less than ϵ, in this case 0.01 m.

**Figure 6:**
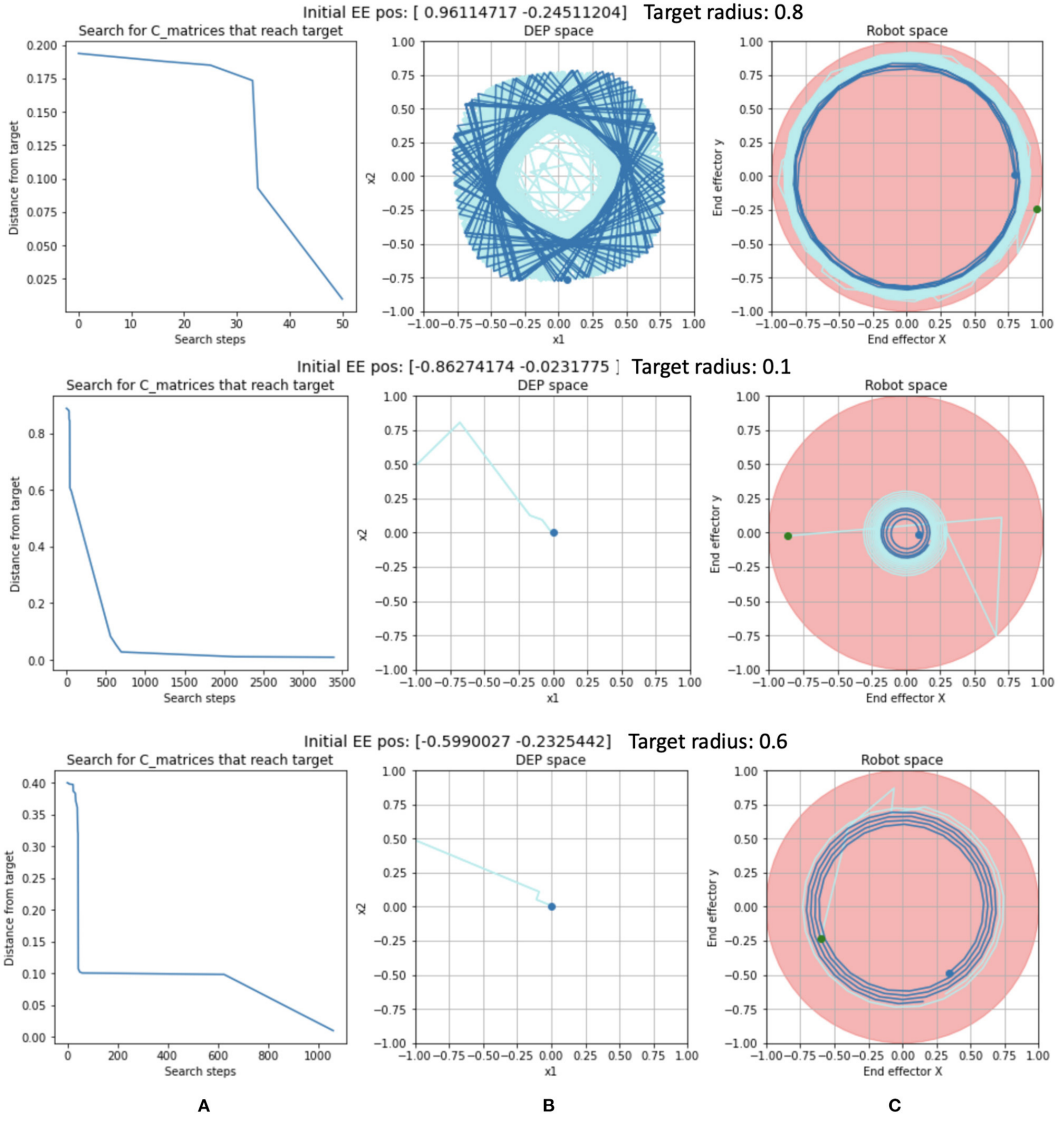
Examples of Circular Trajectory trials with: **(A)** The progress of the search for a solution C matrix. **(B)** The solution trajectory in DEP space. **(C)** The solution trajectory in robot space. The most recent positions are shown in dark blue while the early part of the trajectory is in light blue.

Trajectory examples are shown in Figure 6. In the example in the top row, the search algorithm required a mere 50 steps (Figure 6A) to find a limit cycle in DEP space (Figure 6B) that completed a circle of the desired radius (Figure 6C) after 551 time steps of the rollout.

In the second example, in the second row of Figure 6, the search algorithm required 3,412 search steps (out of a maximum of 4,096) (Figure 6A) to find a solution in DEP space (Figure 6B) that completed a circle after 332 time steps of the rollout.

**Figure 7:**
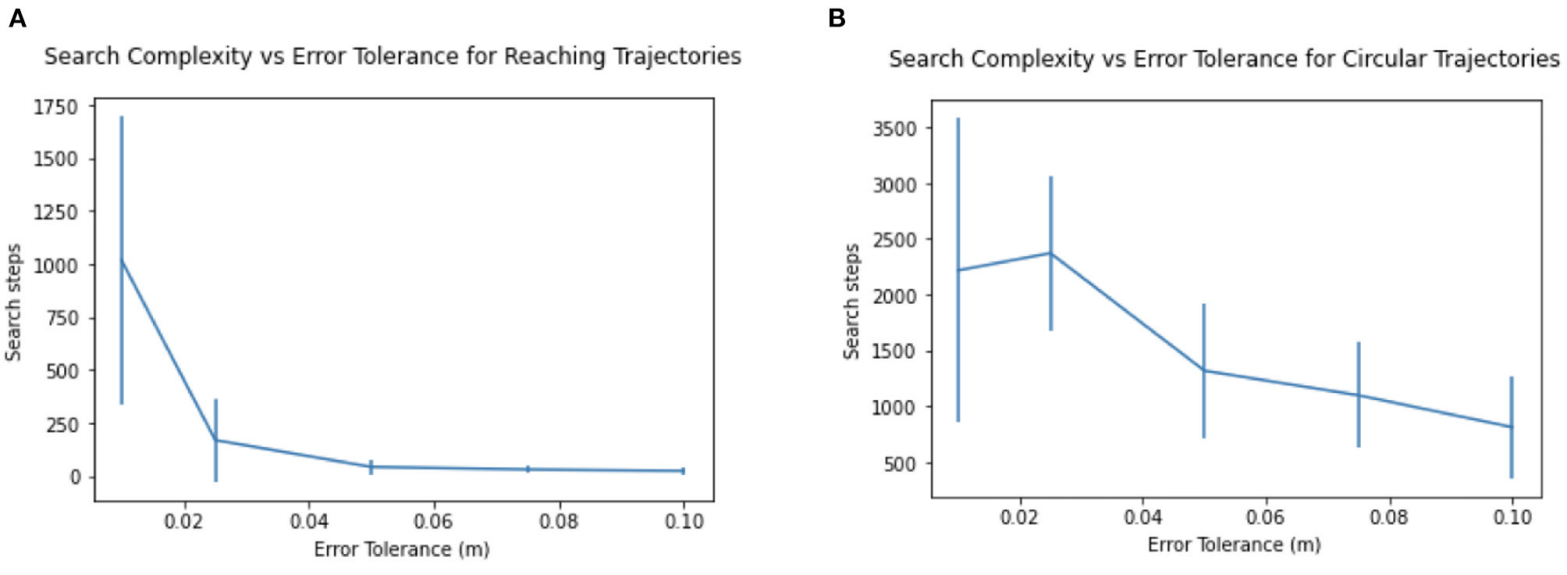
Search complexity and variance increases with lower error tolerance, for **(A)** Reaching trajectories and **(B)** Circular trajectories.

The relationship of search time to tolerance ϵ can be seen in Figure 7. For lower, more stringent, error tolerances ϵ, the number of search steps required increases, as does its variance. Increasing the tolerance required for reaching even slightly (say from 0.01 to 0.025 m) reduces the search steps required by 75%.

## 4. Discussion and Future Work

The controller described in this paper is unlikely to signal the end of inverse kinematics. To borrow Dr. Johnson's phrase, it “is like a dog's walking on his hinder legs. It is not done well; but you are surprised to find it done at all” (Boswell, 1791). Why do these results, and DEP in general, matter? We can answer in three ways.

### 4.1. DEP as a Control Mechanism

First, what is the prognosis for DEP as a control system? The present controller has reduced a high dimensional control problem to one of simple selection of one of 4,096 different discrete values of the *C*_0_ matrix. The original motivation for this paper was to find a way to leverage DEP within the context of Reinforcement Learning. *C*_0_ provides a low dimensional interface for higher level systems to exploit. Yet most of the solutions are indirect, taking time for the end effector to reach its goal.

The search algorithm could be extended to optimize for lower time steps to reach the desired target position or trajectory. Different trajectory types could be produced with different driving functions, although fewer functions would be preferable to more. Driving functions could be abstract, as they are here, or derived from physical models of body elements, such as springs, tissue, or muscles.

There is scope for improving the search algorithm itself from a simple grid search, depending on what patterns, if any, can be found in the mapping of target to *C*_0_. Are there basins of attraction for *C*_0_? Is this controller learnable in a way that generalizes?

Once understanding of the core behavior of “short-circuit” DEP has improved, environmental awareness, one of the core supposed advantages of the neuroplasticity rule, could be reintroduced. This opens the way to recovery from perturbations and short term, “reflex” reactions to changes in the environment.

### 4.2. The Study of DEP

A continuing expressed frustration in the DEP literature is the lack of a full analytical treatment of DEP behavior. That may be due a lack of human resources applied to the problem, or it may be that a full treatment is simply intractable. Some algorithms are mathematically “undecidable,” which is to say that their behavior cannot be predicted without executing the algorithm itself. Perhaps DEP falls into this category.

In either case, this paper follows recent work in taking an empirical, engineering approach to analysing DEP, rather than a theoretical treatment. There remain many questions to be answered.

DEP has produced some fascinating simulations, with realistic looking and intriguing behaviors, such as gait switching, overcoming obstacles, and interaction with devices such as handles. How much of the observed behaviors are due to DEP as a neuroplasticity rule and how much are due to the particular body morphology of the simulated agents? Passive walkers also produce realistic behaviors and respond to the environment in a limited way, yet they have no neuroplasticity at all. Clearly, the agents behavior is generated by the complete system of neuroplasticity plus body plus environment. How we can disentangle the contributions of each?

Finally, does DEP scale? What are the limit cycles of higher dimensional DEP systems? Our understanding of DEP behavior is only just beginning.

### 4.3. Leveraging Pre-existing Complexity

DEP is an example of self-organization in action: of complexity generated from simple rules. Self-organization is easy to spot, but hard to design, yet may be necessary to enable long-term learning processes such as evolution to work effectively (Kauffman, 1995). Classical DEP is a system that, in that evocative phrase, exists “on the edge of chaos,” producing a rich set of behaviors even in the “short circuit” version. Is this complexity useful to agents, or is it a simple artifact?

The leverage of pre-existing complex behaviors is seen in Physical Reservoir Computing (PRC), a field that applies a thin layer of learning over highly complex, pre-existing dynamics in a real or simulated body. A PRC system leverages a set of dynamical behaviors as if they were basis functions and combines them using a shallow artificial neural network. The network can then be trained to perform some desired function. The dimensionality of a problem that might require training a very deep neural network has been reduced to that of training a shallow one.

In the case of PRC, the pre-existing complexity is physical. In other cases it may be algorithmical. A curious example is the history of procedural content generation in computer games (Smith, 2015). The practice originated over 40 years ago with the need to generate details of thousands of planets in highly resource-constrained computers. Rather than store such details, they were generated from the Fibonacci sequence, passed through an interpretive function analogous to our “driver function.” By using a predictable mathematical sequence that has inherent complexity, a vast amount of content could be generated *ex nihilio*. Other examples of Algorithmic Information have been studied, such as the “undecidability” and Turing completeness of Rule 110 (Cook et al., 2004).

What is unclear is whether and to what extent nature has leveraged these potential sources of complexity. In developmental biology, there is a gap between the information specified in the genome and the complexity of the end product (Hiesinger, 2021). In learning there is a gap between the mechanisms we have available and the complexity of the problems to solve. Does pre-existing complexity play a part in closing this gap? Is DEP an example of this?

Differential Extrinsic Plasticity remains a fascinating phenomenon. Neuroplasticity remains an under-explored component of Embodied Intelligence and a rich opportunity for future work.

## Data Availability Statement

The original contributions presented in the study are publicly available. This data can be found here: GitHub, https://github.com/SimonBirrell/dep-control.

## Author Contributions

SB researched DEP, performed the experiments, and wrote the paper under the supervision of FI, with suggestions and comments from AA. All authors contributed to the article and approved the submitted version.

## Funding

This project was possible thanks to EPSRC Grant EP/L01-5889/1, the Royal Society ERA Foundation Translation Award (TA160113), EPSRC Doctoral Training Program ICASE Award RG84492 (cofunded by G's Growers), EPSRC Small Partnership Award RG86264 (in collaboration with G's Growers), and the BBSRC Small Partnership Grant RG81275.

## Conflict of Interest

The authors declare that the research was conducted in the absence of any commercial or financial relationships that could be construed as a potential conflict of interest.

## Publisher's Note

All claims expressed in this article are solely those of the authors and do not necessarily represent those of their affiliated organizations, or those of the publisher, the editors and the reviewers. Any product that may be evaluated in this article, or claim that may be made by its manufacturer, is not guaranteed or endorsed by the publisher.

## Footnotes

^1^Using Hebbian learning here would give a system that resembles a continuous variable Hopfield Network, but with normalization and an inverse model.

^2^ https://github.com/SimonBirrell/dep-control

## Supplementary Material

Supplementary Video 1 Evolution of the dynamics in the first row of Figure 5.

Supplementary Video 2 Evolution of the dynamics in the second row of Figure 5.

Supplementary Video 3 Evolution of the dynamics in the third row of Figure 5.

Supplementary Video 4 Evolution of the dynamics in the first row of Figure 6.

Supplementary Video 5 Evolution of the dynamics in the second row of Figure 6.

Supplementary Video 6 Evolution of the dynamics in the third row of Figure 6.
